# Next generation diabetes scientists shape global research culture: A reflective proposal from postdoctoral researchers in diabetes research

**DOI:** 10.1111/apha.13455

**Published:** 2020-05-20

**Authors:** Attendees of the Danish Diabetes Academy Winter School 2019

## 1 INTRODUCTION AND CONTEXT

The annual Winter School of the Danish Diabetes Academy (DDA) in November 2019 challenged postdoctoral researchers with tough questions regarding research culture that scientists around the world are discussing. The complexity and competitiveness of modern research makes it increasingly difficult for junior researchers to navigate in the science community. This editorial reflects the voices of nearly 200 international researchers ranging from early‐career scientists to professors and medical doctors discussing five challenges of modern research culture—and proposes innovative solutions to overcome them.

Some of the challenges that we, as a research society, are facing today can be condensed into the following overall categories: assessing researchers, securing funding, publishing unbiased data, accessing data and research results by open databases, and increasing the public's trust in science.^1^ Collectively, these represent core aspects of “research culture”—a global term that represents the norms, values, expectations, attitudes, and behaviors of the scientific community.

What does ‘succeed’ mean? What is ‘good science these days? And how do we survive in this hyper‐competitive environment? It is time to rethink the way our culture operates within research, in order to make the most of our resources, time and knowledge and to encourage rather than scare away junior talents from pursuing a career in science.

## 2 CHALLENGE 1: HOW SHOULD RESEARCHERS BE ASSESSED? HOW CAN WE REWARD GROUPS AND COLLABORATIVE EFFORTS?

Producing original research is an expected and rewarded activity among academics. A central criterion of researcher assessment, including for employment, promotion or tenure, focuses mainly on number of publications, publication impact and funding. Authorship of publications is often biased and does not accurately reflect the authors' contributions. This concern has never been as evident as today, since ‘team science’ is not only increasingly more popular and efficient but often required for high‐standard interdisciplinary science, resulting in publications featuring multiple authors. Team science and collaborative efforts can be a golden road to scientific discovery and care must be taken to implement multifactorial assessment systems that reflect the changing research environments and give appropriate credit to all parties involved.

### 2.1 Solution: A multifactorial researcher assessment platform

To offer a fairer system that takes into account a wide range of valuable professional skills, we propose a new multifactorial metric system for researcher assessment. Instead of focusing solely on authorship and funding this system will aim to incorporate a plethora of skills, necessary for scientists at any career stage. Useful throughout the scientific community, this system should include several essential skills and outputs for researcher assessment such as publications, supervision, mentorship, teaching, public outreach activities, funding/grant contributions, specialized skills (eg, statistics, programming, language, research methodology, clinical), collaborative skills (eg, peer review, formal collaboration, technical/protocol support), project management skills, etc For each of the skills an assessment score will be calculated, and an overall score will be provided for each individual researcher. In addition to guiding an individual researcher's career development, the system could also be used to build stronger teams, matching up scientists according to their given skill sets thereby enriching team diversity, maximizing the use of individual members' strengths and enabling them to learn from each other. In order to translate this plan into action, funding would be needed for the development and testing as well as ongoing monitoring and management of validity as a universal tool for researcher assessment. However, only with support from the scientific community would the system be useful, where it could be required in job applications or grant proposals, similar to the use of the ORCID.

Until such a researcher assessment system is available and widely used and to further improve current practices in assessment, ensuring that accurate credit is correctly attributed to a researcher for their work, we recommend that changes be implemented in the authorship system.^2^ Existing systems, such as the CRedIT^3^ and Octopus^4^ could be utilized or incorporated for this purpose.

## 3 CHALLENGE 2: HOW SHOULD FUNDING PRIORITIES BE SET IN SCIENCE?

Fundraising is a ubiquitous concern for scientists throughout their careers. Resources tend to be granted to ‘hot’ topics and/or scientists who have been funded previously, creating a hyper‐competitive environment, with a very low success rate, whereby funding for junior scientists is diminished, as available funds are swallowed up by ‘big fish’ grant receivers. It is probably not surprising that more funding generates higher quality science, whereas increasing competition for funding does not necessarily increase the quality of the proposed projects.^5^ However, the lack of funding is a huge challenge that restricts the launching of new projects. Even though it seems hard to influence resource amount or change a well‐established system, we propose simple action plans, which could provide an improved distribution of funding granted.

### 3.1 Solution 1: Separate funding opportunities for early‐career and well‐established scientists

As it is now researchers at different stages often apply for the same type of grants. To get a fairer distribution between junior and senior grant receivers funding agencies could separate grant applications from junior and senior researchers more than at present. This would increase the chances of launching novel research programs as well as promote careers of junior researchers. Such a model has already been employed by several funding bodies such as the British Heart Foundation or the Novo Nordisk Foundation, whereby funding opportunities are divided into specific categories, ie junior fellowships, international fellowships, start‐up grants for young investigators or bigger project grants for established principal investigators. Additionally, funding could be even better separated into categories ranging from basic research to translational and clinical research. This approach could remove some of the competition created between disciplines and prevent “hot topic” projects from overshadowing smaller ‘high‐risk high‐gain’ projects proposed by early‐career scientists or new group leaders. Foundations could likewise implement an initial (applicant) blinded review process, shifting the focus from the applicant's name towards the project's methodology and impact of proposed research. Lastly, more transparency in the evaluation process, with better clarification of the scoring system and more thorough or even guaranteed feedback on rejected applications would allow scientists to spot their weaknesses (eg, not enough preliminary data to support hypothesis, poor methodology, poor research impact) and improve for future applications.

### 3.2 Solution 2: Funding teams not individuals

With the growing interdisciplinary nature of research, another solution could be to increase support to research teams rather than grant support only to individuals. Currently, successful grant applications often include collaborations of different laboratories with mixed scientific expertise; however, still a great part of the evaluation relies on the applicants' publication track record. Thus, using an individual evaluation approach could mean that talented junior researchers, naturally with fewer publications early in their careers, would be neglected. Awarding teams would not only unite people with different backgrounds, but also broaden networks and exposure to diverse skill sets within a research team. Finally, considering the global nature of science, in addition to funding teams and consortia in a single country/location, funders need to be open to supporting teams in different countries/locations to carry out the same projects.

## 4 CHALLENGE 3: HOW DO WE INTRODUCE A POSITIVE INFLUENCE OF FAILURE AND RAISE THE VALUE OF NEGATIVE RESULTS FOR THE SCIENTIFIC COMMUNITY?

Studies with successfully proven hypotheses are more often considered worthy of publication than studies confirming the ‘null hypothesis’ and are vastly represented in the scientific literature. Hence, 85% of original data papers on PubMed are ‘positive‐data‐biased’.^6^ This publication trend is in contrast to the large quantity of results that confirm the so called ‘null hypothesis’ which scientists deal with on a day‐to‐day basis. Such studies can be viewed as boring for journals, a costly waste of resources, and even a culprit of ruining one's career as a researcher, collectively causing an enormous demotivation among junior scientists. Under‐reporting and filing away of negative results significantly impact science and research culture by skewing the view of reality.

### 4.1 Solution 1: A positive spin on negative data

Embrace your negative data: they force us to think; they drive honest science and are extremely valuable to the scientific community. A cultural change in how ‘null studies’ are perceived by scientists is necessary: Negative data are not a failure. They are as important as positive data and an integral part of scientific progress. As a scientific community, we should revisit the terminology of ‘null studies’ or ‘negative data’ and refer to these with a less negative connotation. Indeed, negative data are often positive to scientists, ie, when side‐effects of new therapeutic compounds are examined. We therefore, propose that the following model should be adapted and encouraged: formulation of hypothesis→generation of results→publication of unbiased results. We should not only aim to publish, but also to cite negative data, acknowledging that a way to change the culture is to start by changing the way we view these findings and how we handle and talk about null studies.

### 4.2 Solution 2: A method‐based, rather than result‐based peer review

Publishing null studies in scientific journals would be supported by an unbiased review process based on an evaluation of the methods used to address hypotheses, as well as the novelty of the findings. This includes a thorough examination of experimental study designs and rigor of statistical analyses. A method‐based review process would ensure that weight and significance would be distributed similarly among studies with negative and positive data. Further examples of method‐based research are resource papers using ‘omics’ approaches, eg, reporting proteome or transcriptome data revealing unbiased and large sets of proteins or genes altered by a pathophysiological condition. Finally, inclusion of raw data packages provides a more impartial and unbiased interpretation of results.

## 5 CHALLENGE 4: HOW DO WE MAKE OPEN SCIENCE PRACTICES MORE APPEALING TO SCIENTISTS?

Open science, the movement to make research protocols and data accessible to all, bears a magnitude of benefits, such as creating trust in the scientific community, sharing of resources and increasing data re‐usability. However, despite these conspicuous benefits, researchers are still reluctant to share their knowledge, their ‘know‐how‐to’ or ‘know‐how‐not‐to’ and unpublished discoveries. Major challenges to open science are restrictions such as data ownership, intellectual property rights, political and economic positioning as well as lack of a clear reward system that would promote open science practices.

### 5.1 Solution 1: Promote education about open science and its benefits

The ease of information sharing via online clouds or social media has revolutionized virtually all fields in the 21st century, and has become an essential part of our everyday lives. Yet, when early‐career scientists are asked ‘What is open science and what are the benefits of sharing your data?’—half of the crowd nods uncomfortably and only a handful are aware of what the term entails.

Several universities in the US and Europe have now launched new programs with information services, guidelines and comprehensive information in order to foster open science into scientific practice. Their offices offer consultation appointments for scientists to discuss strategies of pre‐publishing scientific hypotheses or databases. For example, the University of Helsinki strongly promotes education about open science, open data and open access via web pages such as ThinkOpen,^7^ with the largest percentage of open publications (58.5%) published in one of Helsinki University faculties in 2018.^8^ Education about the open access journals, repositories of journals, code and data (eg, ResearchGate, GitHub, GEO) as well as preprint archives discussed below, should be promoted as routine practice for open science. Collectively, education for everyone in academia about the significant opportunities in open science should broaden the output of research.

### 5.2 Solution 2: Reward scientists who share their data

Evidently, increasing awareness and promoting education about open science via training programs at universities, eg, as part of PhD modules, scientific conduct training or managerial training, would benefit junior researchers as well as more senior scientists and promote good practice towards a more solid scientific integrity.

Publishing data articles in scientific journals or online databases is a means of data sharing, yet it comes with a cost, and it is somewhat time‐ineffective due to the lengthy peer‐review process. Although peer review is necessary, a published draft before peer review, or the so‐called ‘preprint’, could increase web data traffic and promote journal loyalty, aiming to make scientific data more accessible to propagate knowledge faster. There are substantial amounts of data out there, yet the reward systems for open science practices are not yet flourishing. Introducing citations of manuscripts or databases published in preprint archives such as bioRxiv^9^ and including them in researcher assessment systems would ensure a timelier availability of scientific data compared with the typically lengthy review process required by medical journals.

Funding bodies should also encourage or even require openness and pre‐publishing of data or research project plans. Finally, the promotion and adoption of open science could be incorporated into the ranking criteria of universities – in order to create role models in research communities.

## 6 CHALLENGE 5: HOW DO WE IMPROVE THE PUBLIC'S TRUST AND PERCEPTION OF SCIENCE AND AVOID MISINTERPRETATION OF SCIENCE IN THE NEWS?

There is a disturbing increase of distrust in the public debate about science. A Danish survey revealed that 82% of the 1007 Danish citizens participating in the survey only vaguely (or not at all) trust that the media report scientific findings truthfully; however two out of three respondents have a high or very high degree of trust in researchers.^10^ Too often miscommunication and the need for sensational news lead to this outcome. So how can we as scientists avoid media misinterpretation?

### 6.1 Solution: Communication and education are key!

A key to establishing public trust in news outlets covering scientific findings is through public education with a focus on the basics of scientific research, statistics, and critical thinking, from primary school and throughout the school system. A course covering the history of health science could be implemented to emphasize the success of modern scientific research and, eg, how it has reduced morbidity, improved life expectancy and overall quality of life.

In addition, educating scientists in public engagement and collaboration with journalists on communicating science news to the public is an important factor. Improving scientists' engagement in public communication could be achieved in a variety of ways, eg, through a mandatory PhD course or certification on top of the PhD, teaching the doctoral researcher how to communicate their science as part of their training program. Furthermore, the faculties could host regular “open house” or “science cafe” events for the public, inviting them to visit laboratories and meet the scientists behind the research, a method already employed at some universities. On the institutional level, a science communication center, providing expertise on demand, could likewise be established to support the scientists in public communication. Additionally, general training of journalists on how to interpret and accurately report scientific findings is of paramount importance to avoiding misinterpretation. A survey from Reuters Institute for the Study of Journalism, revealed that more than half of online users get their daily news via social media platforms,^11^ thus the moderators of such platforms share the responsibility to curb the flow of misleading and/or fake news to the public.

## 7 CONCLUDING REMARKS

This editorial was created in order to inspire the science community on new ways to improve the research culture we operate in on a daily basis, not only in the field of diabetes, but as common challenges faced by many in the science community. It should be noted that the solutions presented here are not final answers, but hopefully an aide in the ongoing work to improve research culture. Likewise, we emphasize that change in research culture is a task for all scientists, one that requires changes in our own mindset as well as in the mindset of our stakeholders.

The scientific community is a gold mine of potential—potential that we are still striving to extract. A collective shift in our cultural mindset as scientists can help us to unfold the full capacity of our research.

*The specific challenges described and the individual solutions suggested herein are based on the work of researchers in the field of diabetes research, therefore, this editorial is notably authored by the ‘Attendees of the Danish Diabetes Academy Winter School 2019’.*

## CONFLICT OF INTEREST

There is no conflict of interest to declare.

## AUTHOR CONTRIBUTIONS

**Challenge 1:** M. M. Broadley (0000-0003-4408-6304), A. Gonzalez‐Franquesa (0000-0001-9081-753X), A. Jonsson (0000-0003-1252-8673), C. B. Christiansen (0000-0001-9496-801X), G. D. Carrasquilla (0000-0002-7147-9421), A. Mamidi (0000-0001-7953-7991), S. M. Ghiasi (0000-0001-8526-8513), H. B. Juel (0000-0002-5763-8545), S. Falk (0000-0003-3245-1845), M. S. Isidor (0000-0003-3396-5389).

**Challenge 2:** P. Aldiss (0000-0002-1809-2092), L. Gillberg (0000-0003-0588-1663), K. Carolo dos Santos (0000-0002-0775-7853), R. Sabaratnam (0000-0002-4085-1083), L. O. Huang (0000-0001-7592-0942), J. S. Quist (0000-0001-6036-0962), J. R. Knudsen (0000-0002-5471-491X), S. Poulsen (0000-0001-9341-1498), R. Quaranta (0000-0002-8365-4952).

**Challenge 3:** M. Tozzi (0000-0002-2576-0183), R. B. Mikkelsen (0000-0002-4848-5743), M. K. Andersen (0000-0001-8227-1469), M. Dall (0000-0002-8577-3162), A. B. Møller (0000-0003-4868-3982), M. H. Drag (0000-0002-7412-6402), S. Panahi (0000-0001-7920-0766), C. L. Lyons (0000-0001-9339-0295), L. Small (0000-0002-9767-9464).

**Challenge 4:** A. Altıntaş (0000-0002-8932-0160), P. Poursharifi (0000-0002-7804-4359), B. Osborne (0000-0002-0197-3010), A. K. Sarvari (0000-0003-4000-0569), L. W. Johnston (0000-0003-4169-2616), M. H. Solheim (0000-0002-6027-0163), I. M. Modvig (0000-0002-6824-2802), A. S. Husted (0000-0002-0079-3923).

**Challenge 5:** N. Z. Jespersen (0000-0002-3488-1657), E. L. Brown (0000-0001-6766-2007), E. Bak (0000-0002-8620-2804), A. Peluso (0000-0002-8653-2248), F. Finger (0000-0002-0763-6250), K. V. Grunddal (0000-0001-9352-7220), K. Rupar (0000-0002-8200-9484), H. T. Vistisen (0000-0003-4929-943X), J. B. Henningsen (0000-0002-3657-3711), T. Ma (0000-0003-2570-9449).
